# Airborne ultrasound pulse amplification based on acoustic resonance switching

**DOI:** 10.1038/s41598-022-23277-8

**Published:** 2022-11-02

**Authors:** Yuki Hashimoto, Yasuaki Monnai

**Affiliations:** 1 Department of Applied Physics and Physico-Informatics, Keio University, Yokohama, 223-8522 Japan; 2 Research Center of Advanced Science and Technology, The University of Tokyo, Tokyo, 153-8904 Japan

**Keywords:** Electrical and electronic engineering, Acoustics

## Abstract

Airborne ultrasound radiation pressure, a nonlinear effect that appears as a static force in mid-air in the presence of strong ultrasound, has recently been applied in novel scientific and industrial fields. However, the output power of an ultrasound transducer remains low mainly due to the significant mismatch in acoustic impedance between a solid diaphragm and air. To circumvent this fundamental challenge, we propose to emit amplified airborne ultrasound pulses by instantaneously releasing stored acoustic energy into free-space. Specifically, we implement an acoustic cavity with a mechanically rotating shutter covering its open top. Once the acoustic cavity is fully charged, the stored energy is released by opening the shutter. By developing a choke structure that reduces leakage of the stored energy, we generate ultrasound pulses with 2.5 times higher peak power than the input continuous waves at 40 kHz. This preliminary result has a great potential to generate high-power ultrasound pulses using a conventional air-coupled transducer by separating the storage and radiation process, thus circumventing the fundamental limitation brought by impedance mismatch.

## Introduction

The use of airborne ultrasound radiation pressure has recently been discussed in a variety of emerging applications including non-contact manipulation in chemistry and biology^1–3^, levitation of macroscopic objects^4–7^, particle transportation^8,9^, mid-air image projection^10^, and tactile feedback^11–14^ . These applications are based on ultrasound radiation pressure, a nonlinear effect that appears as a static force in mid-air in the presence of strong ultrasound^15–17^. Despite the increasing demands for high-power airborne ultrasound transducers, the output power of conventional air-coupled piezoelectric transducers is limited mainly due to the significant mismatch in acoustic impedance between a piezoelectric material ($\sim 3\times 10^7 \mathrm {kg/m^2 s}$) and air ($\sim 410 \mathrm {kg/m^2 s}$), preventing efficient kinetic-to-acoustic energy conversion^18^. To circumvent the impedance mismatch of the piezoelectric transducers, techniques of airborne ultrasound generation using electrostatically actuated soft diaphragms have been considered^19–21^. However, such capacitive transducers generally have less tolerance for high-voltage application due to the lower electric breakdown threshold, which limits the output power. As an another approach, thermo-acoustic emission of airborne ultrasound from porous silicon has also been proposed^22^. While the non-resonant mechanism allows broadband operation, the thermal-to-acoustic conversion efficiency is a limiting factor of the output power. It should also be emphasized that there have been intensive studies to amplify ultrasound signals. Such amplification has been observed in piezoelectric materials, in which the acoustic propagation couples to electrically driven charge carriers^23–27^. More recently, the development of phonon laser, which is analogous to the light laser but emits phonons instead of photons, has been demonstrated^28–33^. Also, intense ultrasound focusing by temporally manipulating a pulse in a dispersive one-dimensional phononic crystal waveguide has been presented^34^. However, these amplification methods all rely on physical processes in a solid medium and are not suitable for amplifying airborne ultrasound.

**Figure 1:**
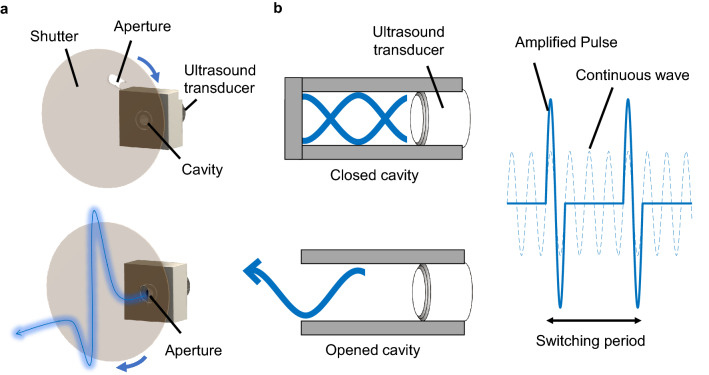
(**a**) Concept of airborne ultrasound pulse amplification using a switchable cavity. (**b**) Illustration of the inside of the cavity when closed and opened. When the stored energy is released into free-space as a pulse, the peak power becomes higher than that of the input continuous waves.

Here we show an approach to amplify airborne ultrasound by storing and releasing acoustic energy using a switchable cavity. As a proof of concept, we prepare an acoustic cavity that is covered with a switchable shutter and excited by a commonly available piezoelectric transducer at 40 kHz (Fig. 1a). When the shutter closes the aperture, the Q-factor increases and the acoustic energy is stored in the cavity. Once the cavity is fully charged, the shutter releases the energy into free-space to generate a strong pulse (Fig. 1b). We experimentally demonstrate that the peak power of the released pulse becomes 2.5 times higher than that of the input continuous waves. We expect that further optimization of the input acoustic impedance and the closed Q-factor of the cavity will enhance the amplification. Although we use a rotating shutter in this study to switch the cavity, there are recently emerging acoustic switches based on an artificial medium^35,36^, electrostatic control of acoustic transparency^37^, and dynamic control of acoustic impedance^38^. It will be possible to replace our shutter with those techniques, enabling a higher degree of system integration.

**Figure 2:**
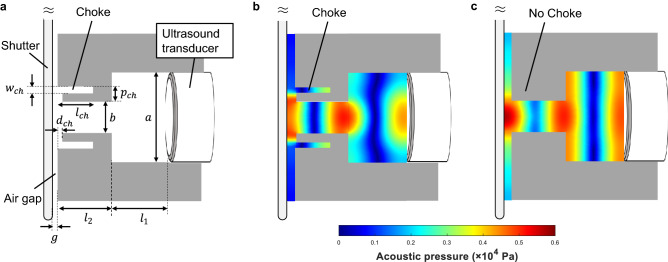
(**a**) Cross-section of the acoustic cavity including the choke structure. (**b**) Simulated acoustic pressure at 40 kHz with the choke structure. (**c**) Simulated acoustic pressure at 40 kHz without the choke structure.

**Table 1:** Design parameters of the cavity shown in Fig. 2a.

Parameter	Value (mm)
*a*	16
*b*	5.4
$l_1$	4.4
$l_2$	4.2
*g*	0.5
$w_{ch}$	0.7
$p_{ch}$	2.6
$l_{ch}$	3.2
$d_{ch}$	0.1

**Figure 3:**
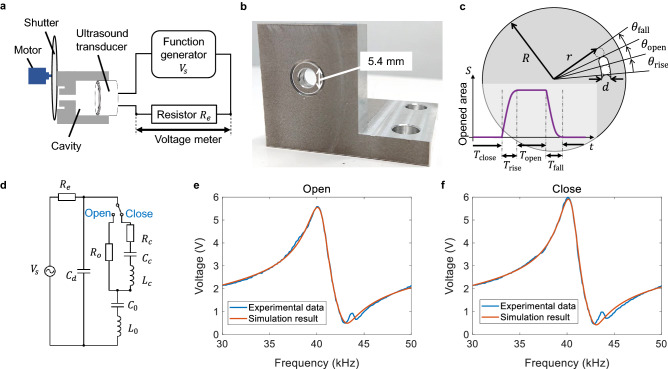
(**a**) Schematic of the cavity combined with the shutter and the transducer. The frequency response of the system can be measured by monitoring the voltage across the external resistor $R_e=463\,\Omega$ for different frequencies. (**b**) Photograph of the fabricated cavity made of aluminum. (**c**) Definition of the shutter geometry. (**d**) Equivalent circuit model of the switchable cavity. The AC voltage source is kept at 10 Vpp (5 V in amplitude) throughout this study. (**e,f**) Measured and calculated frequency response of the voltage across the external resistor $R_e$ when the shutter is opened (**e**) and closed (**f**).

**Table 2:** Estimated values of the equivalent circuit parameters in Fig. 3d.

Variable	Value
$C_d$	2.0 nF
$C_0$	0.27 nF
$L_0$	$58\,\hbox {mH}$
$R_o$	$420\Omega$
$R_c$	$360\Omega$
$C_c$	7.7 nF
$L_c$	$2.0\,\hbox {mH}$

**Figure 4:**
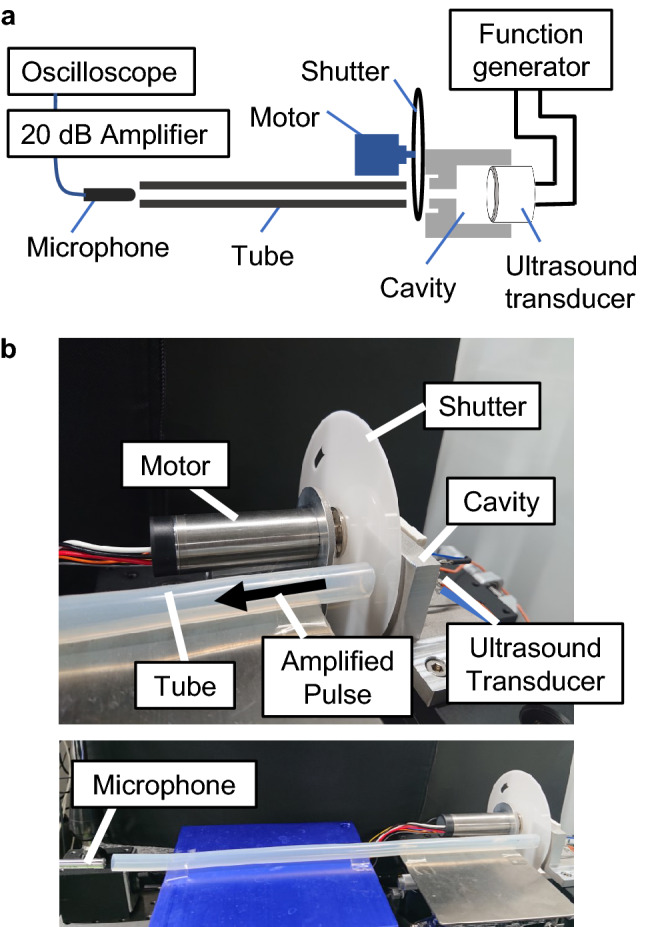
(**a**) Schematic of the experimental setup. (**b**) Photo of the experimental setup.

## Results and discussion

### Acoustic cavity design

We design a switchable acoustic cavity at 40 kHz as illustrated in Fig. 2a. The distance between the closed shutter and the diaphragm of the transducer is set to one wavelength at 40 kHz, i.e., $l_1+l_2=8.6$ mm so that acoustic resonance is sustained. To allow mechanical rotation of the shutter, there is a gap *g* between the open top of the cavity and the shutter. To prevent leakage of the stored acoustic energy through the gap, we develop a choke flange structure, which is a technique widely used in microwave engineering in order to suppress leakage through the gap at a waveguide connection^39^. Specifically, we implement an axially symmetric L-shaped groove surrounding the open top of the cavity as illustrated in Fig. 2a. The leakage is effectively suppressed when the bending point of the L-shaped groove corresponds to a node of the standing wave formed inside the groove. We have also optimized the geometric parameters of the choke $p_{ch}, l_{ch}, d_{ch},$ and $w_{ch}$ as summarized in Table 1. These parameters are determined to maximize the input acoustic impedance at the entrance of the choke based on finite element analysis using COMSOL Multiphysics (See Supplementary Fig. 1). We numerically compare the acoustic fields with and without the choke in Fig. 2b,c, respectively. With the choke, the acoustic power leaked through the gap reduces to 16% of that without the chock, allowing more energy to be stored. Thus, we confirm that the choke effectively prevents leakage. The designed cavity was then fabricated by mechanically processing an aluminum block. A piezoelectric ultrasound transducer with an external diameter of 16  mm and a resonance frequency of 40 kHz is plugged into the open bottom of the cavity. A schematic and a photograph of the cavity are shown in Fig. 3a,b, respectively. The aperture on the shutter is defined as illustrated in Fig. 3c, in which $R =$ 49 mm, $r =$ 37.5 mm, $d =$ 5.4 mm, $\theta _{\mathrm{rise}}=\theta _{\mathrm{fall}}=2\sin ^{-1}(d/2r)=8.3^\circ$, and $\theta _{\mathrm{open}}=12^\circ$ (an explicit formula to calculate the temporal change of the shutter opening area is provided in Supplementary Eq. (10).

To evaluate and analyze the ultrasound amplification, we develop an equivalent circuit model of the acoustic cavity shown in Fig. 3d. We determine the parameters in the circuit as listed in Table 2 based on least squares fitting of the experimentally observed frequency response of the voltage across an external resistor $R_e=463\,\Omega$. The fitting results are shown in Fig. 3e,f, showing agreement except for the small discrepancy in the vicinity of the anti-resonance frequency around 44 kHz, which is probably due to parasitic components excluded in the model. We conclude that the Q-factor of the entire oscillation system comprising the transducer and the cavity is increased from $Q_{\mathrm{open}}=$ 35.1 with the shutter opened to $Q_{\mathrm{close}}=$ 42.9 with the shutter closed.

**Figure 5:**
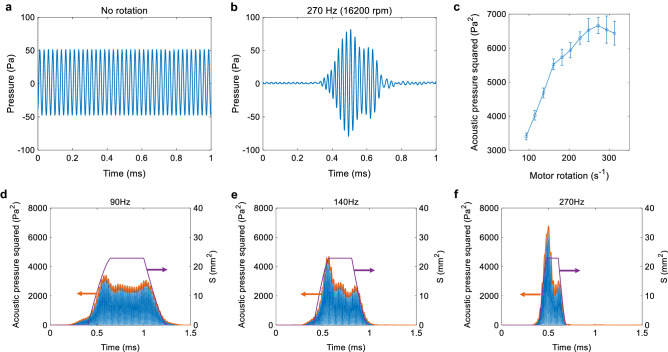
(**a**) Acoustic pressure when the shutter is fixed open with no rotation. (**b**) Acoustic pressure when the shutter is rotated at 270 Hz (16200 rpm). The result is processed by time-synchronous averaging for a measured sequence of 90 pulses. (**c**) Squared peak amplitude of the acoustic pressure as a function of motor rotation speed. Each value is obtained by time-synchronous averaging of 90 pulses of acoustic pressure. Error bars indicate the standard deviation. (**d–f**) Squared acoustic pressure (left axis) and the cavity opening area (right axis) for different rotation speeds of (**d**) 90 Hz, (**e**) 140 Hz and (**f**) 270 Hz.

### Acoustic pressure amplification

We demonstrate ultrasound pulse amplification using an experimental setup shown in Fig. 4a (schematic), b (photograph). When rotating the shutter with a motor, the aperture periodically closes and opens the cavity so that the acoustic energy is stored and released. To fully charge the cavity, the closed state should continue for at least $Q_{\mathrm{close}}/(2\pi ) = 6.8$ times longer than the single cycle of the ultrasound, considering the definition of the Q-factor. That time $T_{\mathrm{charge}}$ can be estimated as $T_{\mathrm{charge}}=Q_{\mathrm{close}}/(2\pi f_u)=170\,\upmu \hbox {s}$, where $f_u =$ 40 kHz is the frequency of the transducer. Similarly, the open state should continue for at least $T_{\mathrm{release}}=Q_{\mathrm{open}}/(2\pi f_u)=140\,\mu \hbox {s}$ to fully release the stored acoustic energy. Meanwhile, considering the design of the shutter in Fig. 3c, the time required to open the aperture $T_{\mathrm{rise}}$, keep the aperture fully opened $T_{\mathrm{open}}$, close the aperture $T_{\mathrm{fall}}$, and keep the aperture fully closed $T_{\mathrm{close}}$ can be calculated as

1 $$\begin{aligned} T_{\mathrm{rise}}&= \frac{1}{f_m} \frac{\theta _{\mathrm{rise}}}{2\pi }= \frac{1}{\pi f_m} \sin ^{-1}\frac{d}{2r} \end{aligned}$$

2 $$\begin{aligned} T_{\mathrm{open}}&= \frac{1}{f_m} \frac{\theta _{\mathrm{open}}}{2\pi } \end{aligned}$$

3 $$\begin{aligned} T_{\mathrm{fall}}&= \frac{1}{f_m} \frac{\theta _{\mathrm{fall}}}{2\pi }= \frac{1}{\pi f_m} \sin ^{-1}\frac{d}{2r} \end{aligned}$$

4 $$\begin{aligned} T_{\mathrm{close}}&=\frac{1}{f_m} \frac{2\pi -(\theta _{\mathrm{rise}}+\theta _{\mathrm{open}}+\theta _{\mathrm{fall}})}{2\pi } \end{aligned}$$

where $f_m$ is the frequency of the motor rotation expressed in Hz rather than rpm. Then, the conditions that effectively store and release the acoustic energy are described as

5 $$\begin{aligned} T_{\mathrm{rise}}&<T_{\mathrm{release}}\le T_{\mathrm{rise}}+T_{\mathrm{open}} \end{aligned}$$

6 $$\begin{aligned} T_{\mathrm{fall}}&<T_{\mathrm{charge}}\le T_{\mathrm{fall}}+T_{\mathrm{close}} \end{aligned}$$

We rotate the shutter to satisfy the conditions above while driving the ultrasound transducer by applying sinusoidal voltage of 10 Vpp (5 V in amplitude) at 40 kHz. We then measure the acoustic pressure emitted from the shutter using a microphone. To reduce undesirable vibration and noise from the shutter and the motor, we transmit the emitted pulse through a hollow silicone tube with an inner diameter of 8.8 mm and a length of 33 cm so that the microphone can be placed at a distance (Fig. 4b). In addition, the measured sequence of 90 ultrasound pulses is processed by means of time-synchronous averaging to exclude a random noise in the acquired data.

Figure 5a shows an acoustic pressure when the shutter is fixed open without rotation. In that case, the acoustic pressure is constant at $51.5\,\hbox {Pa}$ at $40\,\hbox {kHz}$. On the other hand, Fig. 5b shows a result of time-synchronous averaging of the acoustic pressure when the shutter is rotated at 270 Hz (16200 rpm). There, the maximum instantaneous amplitude reaches 81.7 Pa. The increment sufficiently exceeds the effect of the acoustic noise generated when the shutter is rotating (see Supplementary Fig. S2a). Figure 5c shows the square of the maximum instantaneous acoustic pressure as a function of the shutter rotation speed. It demonstrates that the instantaneous acoustic power firstly increases as the rotation speed increases and then saturates at a certain level. This is because $T_{\mathrm{rise}}$ decreases with the rotation speed, and yet $T_{\mathrm{open}}$ is long enough to satisfy Eq. (5). This tendency is observed in Fig. 5d–f, in which transient response of the squared acoustic pressure (left axis) is plotted in comparison to the area of the opening aperture, $S_{\mathrm{area}}(t)$, (right axis) for different rotation speeds (results for other rotation speeds are shown in Supplementary Fig. S3). The explicit form of $S_{\mathrm{area}}(t)$ is given as

7 $$\begin{aligned}{}&S_{\mathrm{area}}(t) = {\left\{ \begin{array}{ll} \frac{\pi d^2}{4}-\frac{d^2\phi (t)}{2} + \frac{d^2 \cos {\phi (t)} \sin {\phi (t)}}{2} &{} (0 \le t< T_{\mathrm{rise}}) \\ \frac{\pi d^2}{4} &{} (T_{\mathrm{rise}} \le t< T_{\mathrm{rise}} + T_{\mathrm{open}}) \\ \frac{d^2\phi (t-T_{\mathrm{rise}}-T_{\mathrm{open}})}{2} - \frac{d^2 \cos {\phi (t-T_{\mathrm{rise}}-T_{\mathrm{open}})} \sin {\phi (t-T_{\mathrm{rise}}-T_{\mathrm{open}})}}{2} &{} (T_{\mathrm{rise}} + T_{\mathrm{open}} \le t < T_{\mathrm{rise}} + T_{\mathrm{open}}+T_{\mathrm{fall}}) \\ 0 &{} (otherwise) \end{array}\right. } \end{aligned}$$

8 $$\begin{aligned}{}&\text {where}\,\phi (t) = \cos ^{-1}{\left[ \frac{2r}{d} \sin {\left( \pi f_m t\right) }\right] } \end{aligned}$$

and explains the rise and fall of the emitted acoustic power. Note that in reality, the acoustic pressure begins to appear 1 ms after opening the aperture due to the sound propagation through the tube, but we plotted the graphs so that the onset timing corresponds to each other for convenience of comparison. We have thus demonstrated experimentally that the peak power of the released pulse becomes 2.5 times higher than that of the input continuous waves. It should be mentioned that while the instantaneous peak power is amplified, the time-integrated emitted energy is decreased in this study because the shutter is closed most of the time during rotation. It will be possible to enhance the repetition rate of the amplified pulse emission by incorporating multiple apertures on the shutter. The maximum number of apertures can be implemented when two adjacent apertures are placed so that $T_{\mathrm{close}}=T_{\mathrm{charge}}-T_{\mathrm{fall}}$ holds as suggested from Eq. (6). At that condition, the time-integrated emitted energy will be close to that of the bare transducer, and the temporal compression of the acoustic energy becomes most efficient.

### Analysis on stored and released energy

We compare the experimentally observed acoustic energy with the theoretically predicted energy stored in the LC circuit modeled by $L_c$ and $C_c$ in Fig. 3d. Since the emitted acoustic power density is given as $p^2/2Z$, where *p* is the acoustic pressure and $Z=410\,\hbox {kg}/\hbox {m}^{2}\,\hbox {s}$ is the acoustic impedance in air, the stored energy emitted from the cavity can be approximately estimated from the experiment as

9 $$\begin{aligned} U_p=\frac{\Delta p^2}{2Z} \frac{T_{\mathrm{release}}}{2}\frac{S_t}{AB} =1.2\times 10^{-7}\,J \end{aligned}$$

where $\Delta p^2$ is the difference of the squared acoustic pressure before (2650 $\hbox {Pa}^2$) and after (6670 $\hbox {Pa}^2$) the amplification (Fig. 5a,b), $T_{\mathrm{release}}=140\,\upmu \hbox {s}$ is the release time of the stored energy and is divided by 2 to account for the nearly triangular change during the pulse emission as shown in Fig. 5f. $S_t=6.1 \times 10^{-5}\,\hbox {m}^{2}$ is the cross-section of the tube, and $A=0.49$ and $B=0.35$ are both experimentally characterized values at 40 kHz accounting for the transmission loss of the silicone tube after traveling 33 cm and the coupling loss with respect to the free-space gap of 5 mm at the entrance (facing the shutter) and the exit (facing the microphone) of the tube, respectively. On the other hand, using the equivalent circuit model in Fig. 3d, we can calculate the acoustic energy stored in the cavity modeled by the series capacitor $C_c$ and inductor $L_c$ as

10 $$\begin{aligned} U_c=\frac{1}{2}C_{c} V^2+\frac{1}{2}L_{c} I^2 = 1.7 \times 10^{-7}\,\hbox {J} \end{aligned}$$

Thus, we confirm that the experimental result in Eq. (9) qualitatively agrees with the analysis based on the equivalent circuit model in Eq. (10). Since the characterization of the transmission loss *A* and the coupling loss *B* required realignment of the tube and the transducer, it was not possible to measure those losses in exactly the same condition as the acoustic pressure measurement. This could explain the discrepancy between the results of Eq. (9) and (10). It should also be noted that while the voltage applied to the transducer was 10 Vpp (5 V in amplitude) in our experiment, higher power is available by applying higher voltages.

## Conclusion

In this study, we have demonstrated a method to generate amplified airborne ultrasound pulses by storing and releasing acoustic energy in a switchable cavity. A choke structure has been designed on the cavity, which prevents energy leakage while allowing free rotation of a shutter. We have experimentally demonstrated that the Q-factor of the system comprising a 40 kHz air-coupled transducer and the cavity can be switched between 35.1 and 42.9 with the shutter. By rotating the shutter at a sufficient speed, we have demonstrated that the peak power of a pulse emitted from the cavity becomes 2.5 times higher than that of the input continuous waves. The result showed qualitative agreement with an analysis based on an equivalent circuit model. The method is compatible with a wide range of already available air-coupled ultrasound transducers. Hence, it will be useful for a variety of applications that utilize airborne ultrasound. The amplification factor will be further increased by optimizing the input impedance and the Q-factor of the cavity. In this study, we have focused on enhancing peak amplitude as it is of particular importance in applications in which a nonlinear effect of acoustic radiation pressure is relevant such as mid-air ultrasound tactile displays and high-speed acoustic manipulation. Although we have used a rotating shutter in this study to switch the cavity, it will be possible to replace it with recently emerging acoustic switches, enabling a higher degree of system integration and faster switching. By developing such faster switching, the duration of the closed state, $T_{\mathrm{close}}$, can be decreased so that the cavity releases the acoustic energy more frequently and negated the reduction of time-averaged acoustic energy emitted in free-space.

## Methods

### Device fabrication

The acoustic cavity shown in Fig. 3b was fabricated by mechanically processing an aluminum block. An ultrasound transducer (SPL Hong Kong, UT1612MPR) with an external diameter of 16 mm and a resonance frequency of 40 kHz was plugged in the open bottom of the cavity. To drive the transducer, AC voltage was supplied from a function generator (NF Corporation, WF1974) with an amplitude of 10 Vpp (5 V in amplitude) at a frequency ranging from 30 kHz to 50 kHz. The shutter was made of polyacetal with a diameter of 98 mm and a thickness of 1 mm. An aperture on the shutter defined in Fig. 3c was formed by drilling. A DC motor (Maxon, ECXSP22L) was used to rotate the shutter at a speed ranging from 5570 rpm to 18780 rpm.

### Determination of the equivalent circuit parameters

We determined the equivalent circuit model parameters in Fig. 3d in the following procedure. We firstly measured the frequency response of the system by monitoring the voltage across the external resistor $R_e=463\,{\Omega }$ connected in series with the transducer. We fixed the shutter so that the cavity was either opened or closed and measured the voltage amplitude across the resistor $R_e$ while sweeping the frequency. Subsequently, we used a circuit simulator (LTspice, Analog Devices) to reproduce the experimental frequency response numerically. We initially determined the parameters $C_d, C_0, L_0,$ and $R_0$ based on a least squares method using the experimental result when the cavity was opened. Then, using those parameters, we determined the other parameters $C_c, L_c,$ and $R_c$ from the experimental result when the cavity was closed. The determined parameters are listed in Table 2.

### Measurement of acoustic pressure

The acoustic pressure was measured with a condenser microphone (Aco, TYPE4157N) connected to a 20 dB amplifier (Aco, TYPE2127). To suppress a noise from the rotating shutter and the motor in the acquired signals, the emitted ultrasound from the cavity was guided by a hollow tube made of silicone rubber with an inner diameter of 8.8 mm and a length of 33 cm. As shown in Fig. 4b, the use of this tube allows the microphone to be placed at a distance from the shutter and the motor. The data was acquired with an oscilloscope (Rigol, DS1054Z) as a voltage sequence of $10^6$ points for 1 s, i.e. a temporal resolution of $1\,\upmu \hbox {s}$. From the sequence, 90 pulses were processed by time-synchronous averaging. The room temperature of the experimental environment was kept at $22^\circ \hbox {C}$.

## Supplementary Information

Supplementary Information.

## Data Availability

The data that support the plots within this paper and other findings of this study are available from the corresponding author upon reasonable request.
