# Supplementary material for: Airborne ultrasound pulse amplification based on acoustic resonance switching
Source: Sci Rep. 2022 Nov 2;12:18488. doi: 10.1038/s41598-022-23277-8 (PMC9630294; doi:10.1038/s41598-022-23277-8)
Supplement: Supplementary file 1 — Supplementary Information. [file 41598_2022_23277_MOESM1_ESM.pdf]

Supplementary Information:

Airborne Ultrasound Pulse Amplification Based on Acoustic Resonance Switching

Yuki Hashimoto[1] and Yasuaki Monnai[2,3]

[1]Department of Applied Physics and Physico-Informatics, Keio University, Yokohama, Japan

[2]Research Center of Advanced Science and Technology, The University of Tokyo, Tokyo, Japan

[3] PRESTO, Japan Science and Technology Agency, Saitama, Japan

Determining parameters of cavity

We determined the design parameters of the cavity (Fig. 2 **a** in the main text) in the following procedure. The main body of the cavity is defined by a, b, l_1, and l_2. The diameter of the open bottom of the cavity $a =$16 mm was determined by the outer diameter of the ultrasound transducer used in this study. The axial length of the cavity $l_1 + l_2 = 8.6$ mm was chosen to be one-wavelength at 40 kHz. To keep the aperture small so that T_{rise} in Eq. (1) becomes short enough and also to leave a space for the choke structure, we set $b = 5.4$ mm and $l_2 = 4.2$ mm (hence $l_1 = 4.4$ mm). The design parameters with respect to the choke structure p_{ch}, l_{ch}, d_{ch}, and w_{ch} were determined as follows. The groove width $w_{ch} = 0.7$ mm was firstly determined by the constraint of the manufacturing tool. We then optimized the combination of p_{ch} and l_{ch} to maximize the average acoustic impedance Z_{ch} at the entrance of the choke

$$Z_{ch} = \frac{1}{S_{ch}} \int_{\text{entrance}} \frac{p}{u} dS \tag{11}$$

where p and u represent acoustic pressure and particle velocity, respectively, and $S_{ch} = \pi b(g + d_{ch})$ is an area of the entrance of the choke defined by axially rotating Fig. 2 **a**. Figure S1 **a** shows the calculated Z_{ch} as a function of p_{ch} and l_{ch}, assuming $g = 0.5$ mm and $d_{ch} = 0$ mm for simplicity. It becomes maximum when $p_{ch} = 2.6$ mm and $l_{ch} = 3.2$ mm. Using these values, we next investigated the dependence of Z_{ch} on d_{ch}. Since Z_{ch} is also dependent on the air gap g, Z_{ch} is averaged for different values g varied from 0.1 mm to 0.9 mm in a 0.1 mm step. The result is shown in Fig. S1 **b**, indicating $d_{ch} = 0.1$ mm leads to the highest impedance. We therefore set $d_{ch} = 0.1$ mm.

Characterization of noise

When the shutter is rotated by the motor, undesirable vibration and noise are generated. We thus used a hollow silicone tube to guide the ultrasound pulse emitted from the cavity so that the microphone can be placed at a distance (Fig. 4 **b**). Here, we show the result of noise characterization, which was measured as an acoustic pressure using the same setup as in Fig. 4 **b** without driving the ultrasound transducer. Figure S2 **a** shows the acoustic pressure measured with the microphone when the shutter rotation speed is 270 Hz (16200 rpm). We also plot its Fourier transform in Fig. S2 **b**, in which we observe spectral peaks at

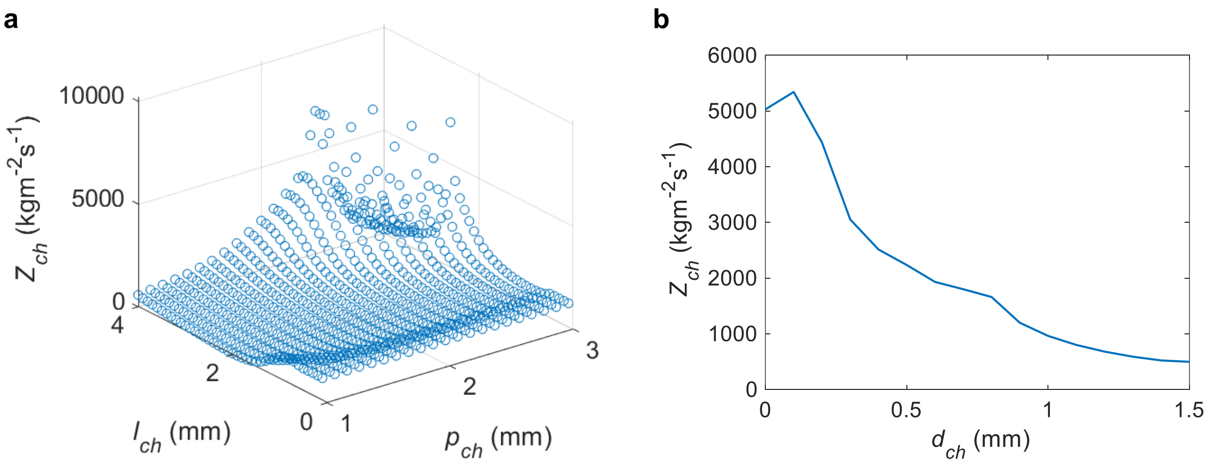

Figure S1. **a** Numerically evaluated Z_{ch} as a function of p_{ch} and l_{ch} when $g = 0.5\,\text{mm}$ and $d_{ch} = 0\,\text{mm}$ are assumed. **b** Numerically evaluated Z_{ch} as a function of d_{ch}. The vertical axis indicates the value of Z_{ch} averaged for different values of g ranging from 0.1 mm to 0.9 mm in a 0.1 mm step.

270 Hz and its harmonics. Thus, we confirm that the periodic peaks in Fig. S2 **a** are attributed to an acoustic noise generated when the shutter opens and closes the cavity even when the transducer is not driven. The peak amplitude at the fundamental frequency as a function of the motor speed is shown in Fig. S2 **c**. We confirm that the noise tends to increase as the rotation speed increases.

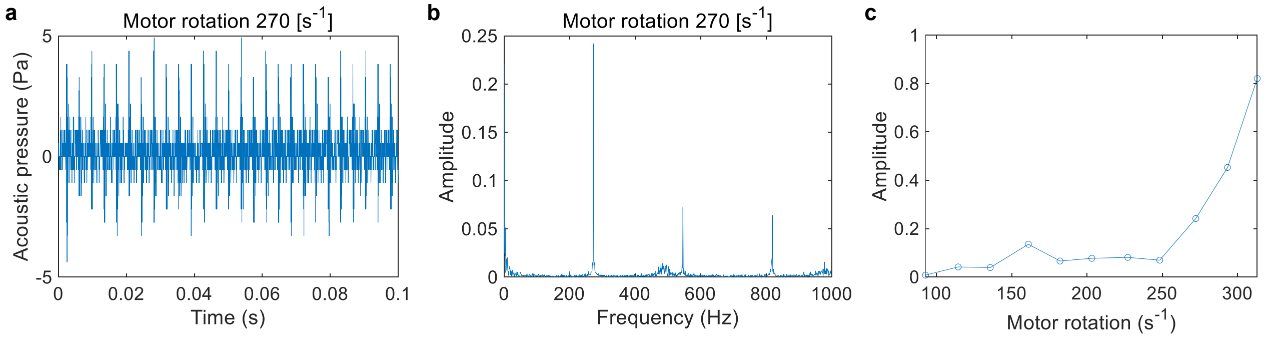

Figure S2. **a** Measured acoustic pressure of the noise generated when rotating the shutter at 270 Hz without driving the transducer. **b** Fourier transform of the noise acoustic pressure in **a**. **c** Peak amplitude of the fundamental frequency of the Fourier transformed noise measured for different rotation speed.

Calculation of shutter opening area

In this section, we show the complete set of the experimental results corresponding to Fig. 5 **d-f** in the main manuscript, in which transient response of the squared acoustic pressure (left axis) is plotted in comparison to the area of the opening aperture, $S_{\text{area}}(t)$, (right axis) for different rotation speeds. Note that in reality the acoustic pressure begins to appear 1 ms after opening the aperture due to the sound propagation through the tube, but we plotted the graphs so that the onset timing corresponds to

each other for convenience of comparison.

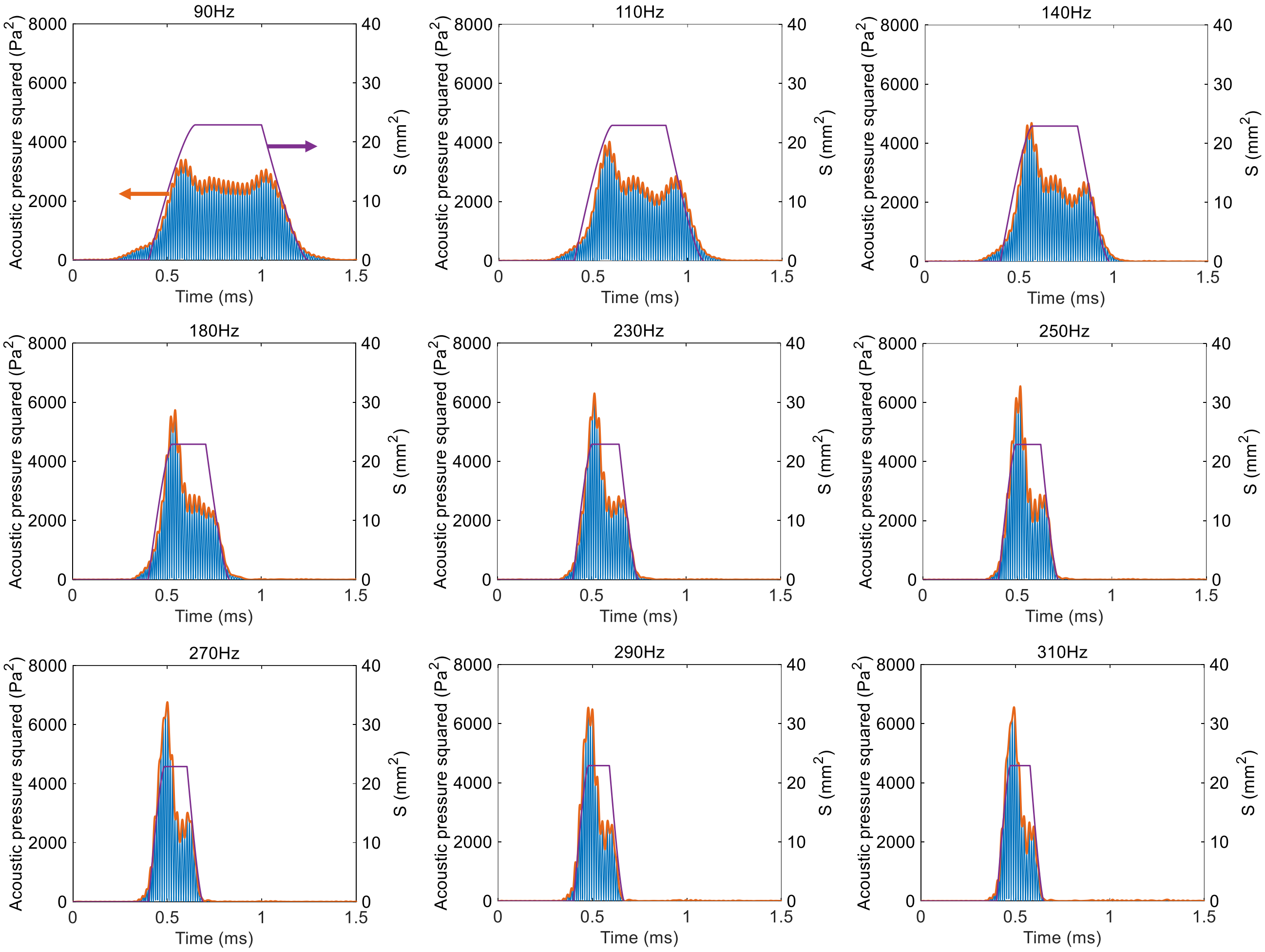

Figure S3. Comparison of the shutter opening area (right axis) and the squared acoustic pressure (left axis) at different rotating speeds. The red lines express the envelopes of the squared acoustic pressure.
